# Book Review

**DOI:** 10.1120/jacmp.v5i3.2067

**Published:** 2004-10-21

**Authors:** Stanley H. Benedict

**Affiliations:** ^1^ Department of Radiation Oncology Medical College of Virginia Hospitals, Virginia Commonwealth University

## Abstract

**Review of Radiation Oncology Physics: A Handbook for Teachers and Students**, edited by Ervin B. Podgorsak, PhD

The opportunity for me to review the textbook *Review of Radiation Oncology Physics: A Handbook for Teachers and Students,* edited by Ervin B. Podgorsak, PhD, could not have occurred at a more opportune time in my career. I am currently teaching my first graduate‐level radiation therapy physics course as part of our newly initiated medical physics graduate program within the Department of Physics at the Virginia Commonwealth University in Richmond, Virginia. Although I have all the notes from my graduate courses in medical physics from UCLA and access to a host of other lectures and syllabi from other institutions, in my opinion this Handbook is ideal for anyone currently teaching a graduate‐level course in medical physics. All the chapters and sections have been very well organized and structured specifically from the viewpoint of presenting lectures on the fundamental concepts of modern radiation therapy physics.

The goal, as stated in the Foreword, is to provide the basis for the education of medical physicists initiating their university studies in the field. It is not designed to replace the large number of textbooks available, which will still be necessary to deepen the level of knowledge in specific topics presented in the Handbook. Rather, the intent is to serve as a factual supplement to the various textbooks on medical physics in the form of a syllabus covering all modern aspects of radiation oncology physics. In addition, the Handbook was developed as a teacher's guide in which the various topics in the syllabus could be expanded to form a detailed “bullet list” that contains the basic guidelines of the material to be included, so that the lectures can be prepared accordingly.

The Handbook has a comprehensive bibliography within each section. I also found that the book successfully fills the gap in the teaching material for the specialty of medical physics, and does so in a single manageable volume with a logical, well‐thought‐out structure for presenting and learning modern radiation therapy physics.

The book has over 530 pages and a host of very well documented figures and tables to supplement the comprehensive listing of topics covered. As an indication of the breadth of coverage, see the titles for the 16 chapters:

1. Basic Radiation Physics
2. Dosimetric Principles, Quantities and Units
3. Radiation Dosimeters
4. Radiation Monitoring Instruments
5. Machines for External Beam Radiotherapy
6. External Photon Beam: Physical Aspects
7. Clinical Treatment Planning in External Photon Beam Radiotherapy
8. Electron Beams: Physical and Clinical Aspects
9. Calibration of Photon and Electron Beams
10. Acceptance Tests and Commissioning
11. Computerized Treatment Planning Systems for External Beam Radiotherapy
12. Quality Assurance of External Beam Radiotherapy
13. Brachytherapy: Physical and Clinical Aspects
14. Basic Radiobiology
15. Special Procedures and Techniques in Radiotherapy
16. Radiation Protection and Safety in Radiotherapy

The genesis of this Handbook is described in the Preamble: in the late 1990s, the International Atomic Energy Agency (IAEA) initiated a systematic and comprehensive plan to support development of teaching programs in medical radiation physics to many of its member states. This effort was aimed at supporting countries to develop their own university‐based medical science programs in medical physics. In that initial effort, they developed a syllabus in radiation therapy physics with a goal to “harmonize” the various levels of training. With the development of the syllabus, a Teacher's Guide was developed in which the various topics in the syllabus were expanded to form a detailed bullet list containing the basics guidelines of the material to be included in each topic so that the lectures could be prepared accordingly. P. Andreo of IAEA led the initial Teacher's Guide, and later, in 2001, E.B. Podgorsak took the lead of the project. Dr. Podgorsak redesigned the contents so that the book is now a comprehensive *Handbook for Teachers and Students* with coverage deeper than a simple guide.

The book was provided to me as a pdf file, which will of course greatly increase its circulation to IAEA member states, in accordance with its original goal. However, navigating a 500+ page document in this electronic format can be difficult. That said, since the Handbook will be provided in this electronic format, it will ideally lend itself to putting some of the text, figures, and tables into slide presentations and thus greatly improve the dynamic visuals of a typical graduate‐level medical physics course. Whereas the Handbook does provide a comprehensive listing of the topics that should be covered in their respective suggested order, it is important to again note that these are not comprehensively introduced, thus necessitating a more traditional textbook to accompany the students’ reading. There are also no example problems and solutions. My main request and suggestion to the authors for future editions is to include, as a supplement, several illustrative example problems and solutions. In my opinion, this well‐organized and well‐structured book, combined with a comprehensive series of example problems, will be a superb and welcome addition to any graduate course in radiation therapy physics.

In summary, *Review of Radiation Oncology Physics: A Handbook for Teachers and Students* is an excellent review book dedicated to students and teachers involved in programs that train professionals for work in radiation oncology. It provides a compilation of facts on the physics as applied to radiation oncology and as such is very useful to graduate students in medical physics programs, residents, dosimetrists, and therapy technology programs. This book is expected to have wide dissemination by the IAEA; however, at this stage, it is published as working material seeking comments, corrections, and feedback.

## Book Availability

The working version of the book is available free of charge. It is a 14‐MB file that can be downloaded from either of two sites. The full book is available from the following site maintained by the editor:

http://www.medphys.mcgill.ca/IAEABOOK/

The full book or individual chapters can be downloaded from the following IAEA site:

http://www‐naweb.iaea.org/nahu/dmrp/syllabus.shtm

